# Sex Differences of the Diabetic Heart

**DOI:** 10.3389/fphys.2021.661297

**Published:** 2021-05-27

**Authors:** Natacha Fourny, Christophe Beauloye, Monique Bernard, Sandrine Horman, Martine Desrois, Luc Bertrand

**Affiliations:** ^1^Pôle de Recherche Cardiovasculaire, Institut de Recherche Expérimentale et Clinique, Université catholique de Louvain, Brussels, Belgium; ^2^Division of Cardiology, Cliniques Universitaires Saint-Luc, Université catholique de Louvain, Brussels, Belgium; ^3^Aix-Marseille University, CNRS, CRMBM, Marseille, France

**Keywords:** type 2 diabetes, cardiovascular diseases, sex differences, gender differences, ischemic heart diseases, personalized care, cardioprotection, diabetic cardiomyopathy

## Abstract

Type 2 diabetes is a chronic disease associated with micro- and macro-vascular complications, including myocardial ischemia, and also with a specific and intrinsic cardiac dysfunction called diabetic cardiomyopathy (DCM). Both clinical and animal studies demonstrate significant sex differences in prevalence, pathophysiology, and outcomes of cardiovascular diseases (CVDs), including those associated with diabetes. The increased risk of CVDs with diabetes is higher in women compared to men with 50% higher risk of coronary artery diseases and increased mortality when exposed to acute myocardial infarction. Clinical studies also reveal a sexual dimorphism in the incidence and outcomes of DCM. Based on these clinical findings, growing experimental research was initiated to understand the impact of sex on CVDs associated with diabetes and to identify the molecular mechanisms involved. Endothelial dysfunction, atherosclerosis, coagulation, and fibrosis are mechanisms found to be sex-differentially modulated in the diabetic cardiovascular system. Recently, impairment of energy metabolism also emerged as a determinant of multiple CVDs associated with diabetes. Therefore, future studies should thoroughly analyze the sex-specific metabolic determinants to propose new therapeutic targets. With current medicine tending toward more personalized care of patients, we finally propose to discuss the importance of sex as determinant in the treatment of diabetes-associated cardiac diseases to promote a more systemic inclusion of both males and females in clinical and preclinical studies.

## Introduction

An alarming report from the International Federation of Diabetes recently highlighted that prevalence of diabetes keeps increasing worldwide, affecting 463 million people in 2019 (International Diabetes Federation, 2019). Cardiovascular (CV) complications remain the predominant causes of morbidity and mortality among diabetic patients with an increased risk of heart failure, coronary artery diseases (CADs), myocardial infarction (MI), diabetic cardiomyopathy (DCM), and stroke (American Diabetes Association, 2015). Despite an estimated prevalence of diabetes slightly lower in women in comparison with men (9 vs. 9.6%) (International Diabetes Federation, 2019), strong evidence suggests worse CV consequences and mortality in diabetic women, independent of age. Consequently, there is a growing interest for a better understanding of the molecular mechanisms involved in this phenomenon (Kautzky-Willer et al., 2016).

## Impact of Estrogens on Cardiovascular System

Multiple studies show that female hormones, particularly estrogens, have a beneficial effect on CV health (Dantas et al., 2012; Kander et al., 2017). Estrogen receptors ERα and ERβ are expressed in endothelial cells, vascular smooth muscle cells, and cardiomyocytes of both sexes (Cid et al., 2002; Iorga et al., 2017). Estrogens can affect lipid metabolism, energy balance, fat distribution, insulin sensitivity, and blood pressure and increase bioavailability of nitric oxide (NO) (Cid et al., 2002; Ventura-Clapier et al., 2017a). Estrogens also positively regulate vascular relaxation factors, such as prostaglandin I2. Therefore, estrogen has the ability to positively regulate CV risk factors, such as obesity, hypertension, and glucose mishandling. Studies on ovariectomized animals demonstrate greater impairment of left ventricular function following an ischemia-reperfusion episode with an implication of apoptosis, pro-inflammatory cytokines, and reactive oxygen species (ROS). Treatment with estrogens resulted in restoration of cardiac function, indicating a potential cardioprotective role of female sex hormones (Lagranha et al., 2010; Yang et al., 2018).

Compared with men, women have a higher percentage of fat mass, primarily accumulating in the subcutaneous area (Power and Schulkin, 2008). Estrogens modulate fat distribution through the expression of their receptors. Of interest, a higher ERα/ERβ ratio has been shown to correlate with lower adiposity, especially at the visceral level (Davis et al., 2013). Importantly, healthy women present lower intracardiac lipid levels than men (Huang et al., 2017), and male sex is a predictor of myocardial steatosis (Kannel and McGee, 1979; Iozzo et al., 2009). Thus, favorable distribution of fat participates in CV health in women. Another important point is the lower blood pressure from adolescence onward, due to 27% less renin system activity (Blenck et al., 2016). Hypertension is a well-known CV risk factor affecting both sexes but with higher incidence and severity in men (Kjeldsen, 2018). Endogenous estrogen maintains vasodilation, contributing to the control of blood pressure in premenopausal women (Garcia et al., 2016).

## Increased Risk in Cardiovascular Complications in Type 2 Diabetic Women

Several parameters linked to sexual dimorphism could contribute to higher CV risk in type 2 diabetic (T2D) women in comparison to T2D men (Table 1).

**TABLE 1:** Sexual dimorphism in cardiovascular risk factors in absence or presence of diabetes.

	**Male**	**Female**	**References**
***Absence of diabetes***			
**Lifestyle**			
Food intake and energy expenditure Risk of T2D with consumption of sugary drinks Physical activity and MI risk Smoking and CADs risk Smoking and diabetes risk	**↑ – ↓ ↓ =**	**↓ ↑ ↑ ↑ =**	Kautzky-Willer et al., 2016 Eshak et al., 2013 Kriska et al., 2006 Thomas, 2017 Willi et al., 2007
**Fat distribution**			
Fat percent Preferential localization Ectopic cardiac fat	**↓** Visceral **↑**	**↑** Subcutaneous **↓**	Power and Schulkin, 2008 Power and Schulkin, 2008; Blüher, 2013 Kannel and McGee, 1979; Iozzo et al., 2009
**Blood pressure**			
Basal systolic and diastolic blood pressure Incidence and severity of HT Cardiac adaptation to HT HF failure risk	↑ ↑ Eccentric hypertrophy **↓**	**↓ ↓** Concentric hypertrophy ↑	Blenck et al., 2016; Kjeldsen, 2018 Anand et al., 2008 Krumholz et al., 1993; Santos and Shah, 2014 Beale et al., 2018
**Glucose metabolism**			
Basal insulin level Risk of diabetes	**↓** ↑	↑ **↓**	Flanagan et al., 2006; Reichelt et al., 2013 International Diabetes Federation, 2019
***Presence of diabetes***			
**Fat distribution**			
Preferential localization Risk of CADs with obesity Cardiac lipid level	Visceral **↓ ↓**	Visceral **↑ ↑**	Power and Schulkin, 2008 Elffers et al., 2017; Lind et al., 2017 Iozzo et al., 2009
**Blood pressure**			
Incidence and severity of HT	**↓**	**↑**	Anand et al., 2008
**Glucose metabolism**			
Manifestation Insulin resistance CV risk with prediabetes	Impaired fasting blood glucose ↓ –	Impaired glucose tolerance ↑ ↑	Rydén et al., 2007 Flanagan et al., 2006; Reichelt et al., 2013 Levitzky et al., 2008

*Arrows in the “male” column indicate differences in comparison to female; and arrows in the “female” column indicate differences in comparison to male. CV, cardiovascular; CADs, Coronary artery diseases; HF, heart failure; HT, hypertension.*

A role of estrogen and its receptors has been evocated to explain the higher CV risk found in T2D women. Increased expression of ERβ compared with ERα is associated with increased oxidative stress, inflammation, and atheromatous plaque formation (Xing et al., 2009), leading to the development of type 2 diabetes and CV complications. Diabetic women present higher insulin resistance (Flanagan et al., 2006; Reichelt et al., 2013) and are more likely to be glucose intolerant, and diabetic men have elevated fasting blood glucose levels (Rydén et al., 2007). Estrogen supplementation in postmenopausal women decreases fasting blood glucose and, thus, improves glucose tolerance (Huang et al., 2017). Ovariectomized Sprague–Dawley females had poorer glucose tolerance than non-ovariectomized animals (Saengsirisuwan et al., 2009). Importantly, prediabetes (fasting blood glucose: 100–125 mg/dL) is predictive of CVDs only in women (Levitzky et al., 2008). The greater insulin resistance found in women, coupled with endothelial dysfunction, may explain the high risk of CV complications in T2D (Rutter et al., 2003).

Importantly, obesity increases the relative risk of CADs by 64% in women as opposed to 46% in men (Elffers et al., 2017; Lind et al., 2017). Besides this, visceral adipose tissue is the source of ectopic deposition of fat in the heart (Tchernof and Despres, 2013), participating in the development of DCM through lipotoxicity (Listenberger et al., 2001; Bugger and Abel, 2014). T2D women have a more pronounced increase in intracardiac lipid content than T2D men (Iozzo et al., 2009). The ERβ receptor prevalence results in an adipogenic and diabetogenic profile (Blüher, 2013; Davis et al., 2013), probably explaining this difference.

Rutter et al. (2003) show that the increase in left ventricular mass and wall thickness correlating to glucose intolerance is more important in women than in men, largely accounted for by obesity and pressure overload. Hypertension is more pronounced in T2D women than in T2D men, and sex appears to influence morphological cardiac adaptation to hypertension (Santos and Shah, 2014). Women tend to develop concentric hypertrophy compared with men who tend to develop eccentric hypertrophy (Krumholz et al., 1993). This is confirmed in animal models (Olsson et al., 2001). A decrease in peroxisome proliferator-activated receptor-α (PPARα) signaling is found in hypertrophic males but not in females (Harrington et al., 2017), and acute inhibition of PPARα blocks the sex difference in hypertrophy development. Accordingly, in humans suffering from aortic stenosis, Kararigas et al. (2014) reveal that cardiac hypertrophy is related to increased activation of profibrotic and inflammatory markers in men compared with women.

## Sexual Dimorphism in Ischemic Heart Diseases Associated With Diabetes

In the general population, incidence of MI remains higher in men than in women. CVDs appear on average 10 years earlier in men than in women (Kannel and McGee, 1979; Thom et al., 2006; Anand et al., 2008; Dantas et al., 2012). Interestingly, women seem to lose this sex-related protection in the presence of T2D (Murphy, 2011). This could be primarily due to differences in diagnosis and treatment of MI itself. Symptoms experienced by women are, in 50% of cases, different from the classic symptoms recognized in men, such as feelings of exhaustion, digestive disorders, and shortness of breath (Mehta et al., 2016), resulting in delayed treatment (Bugiardini et al., 2017). When considering CADs, women have a 50% higher risk than men, presenting increased mortality when exposed to acute MI (Kannel, 1987; Toedebusch et al., 2018) with a strong impact of long-standing diabetes in women (Natarajan et al., 2005). Several studies show a higher risk of CADs at lower glucose levels in women (Koro et al., 2008; Levitzky et al., 2008). The Framingham study also demonstrated that risk of MI is increased by five in T2D women compared with non-diabetic women, and this risk is only multiplied by two in T2D men (Kannel et al., 1974; Wannamethee et al., 2012). Moreover, 38% of women die within 1 year of their first MI although only 25% of men do so (Thom et al., 2006).

Concomitant development of atherosclerosis, endothelial dysfunction, and impairment of the coagulation profile could explain, in part, why diabetic women present a higher risk of ischemic heart diseases (IHDs). Clinical studies reveal a more severe atherogenic dyslipidemia in diabetic women, particularly through an increase in triglycerides and lipoprotein cholesterol concentrations (Walden et al., 1984). Accumulation of oxidized low-density lipoprotein within arteries is a mechanism contributing to the development of atherosclerotic plaques. In particular, Chen et al. (2002) show that its receptor, the lectin-like oxidized low-density lipoprotein receptor-1 (LOX-1), has an important role in atherosclerosis development. Interestingly, sex differences in LOX-1 are reported with a particularly high expression in diabetic and obese women (Brinkley et al., 2008), making it an interesting pathway related to sex differences in diabetes and IHDs.

Atherosclerosis is an important factor contributing to the development of endothelial dysfunction. Diabetic women are also characterized by greater endothelium impairment in comparison to diabetic men. Clinical studies particularly show endothelium-dependent vasodilation alteration, which is confirmed in different animal models of T2D (Alameddine et al., 2015; Ranucci et al., 2019). A decrease in endothelium-dependent and -independent vascular response is observed in female Goto-Kakizaki rats with lower coronary flow and reduced upregulation of the NO pathway (Desrois et al., 2017; Palee et al., 2017). Zhang et al. reveal a predisposition of females to vascular lesions after induction of diabetes in both mesenteric arteries and the aorta (Zhang et al., 2012; Hunter et al., 2017). Regulation of the protein kinase B pathway may also contribute to vascular endothelial dysfunction and myocardial sensitivity to an ischemia-reperfusion episode (Desrois et al., 2004), especially in females (Reichelt et al., 2013). Goel et al. (2008) suggest that estrogen causes gender-specific endothelial dysfunction in hyperglycemic conditions by increasing the expression of PKCβ and increasing O_2_^–^ production in females. Hyperglycemia also alters the balance of estrogen receptors and increases both oxidative stress and the level of vasoconstrictors (Donahue et al., 2007; Wannamethee et al., 2012; Hunter et al., 2017).

**FIGURE 1:**
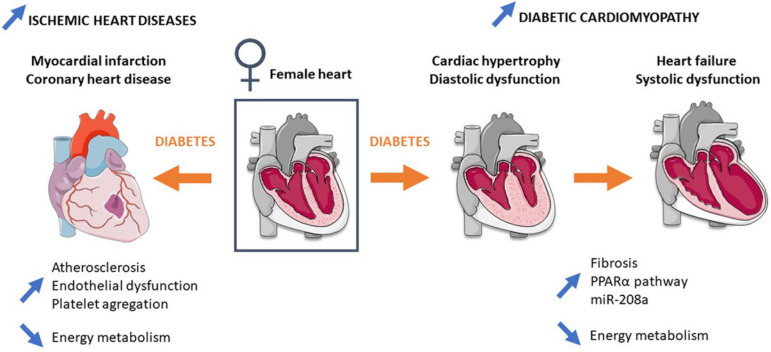
Mechanisms involved in the higher risk and mortality of type 2 diabetic women in ischemic heart diseases and diabetic cardiomyopathy. Detailed information is provided in the main text. Blue arrows represent differences in comparison to diabetic men.

Interaction between endothelial impairment and platelet aggregation is also implicated in atherosclerosis pathogenesis. Diabetic women present elevation of fibrinolytic/thrombotic factors during the transition from normoglycemia to diabetes (Steinberg et al., 2000; Donahue et al., 2007, 2011), leading to a prothrombotic coagulation profile (Steinberg et al., 2000; Donahue et al., 2007). Meigs et al. report an increase in circulating levels of thrombosis-promoting factors (Plasminogen activator inhibitor-1, von Willebrand factor) and adhesion molecules (vascular cell adhesion molecule 1, intercellular adhesion molecule 1) associated with atherosclerosis and microvascular diseases (Meigs et al., 2006; Madhu, 2010). In addition, women with T2D are more sensitive to changes in coagulation and inflammation than men, which could be explained by the fact that women have a larger platelet count as well as higher platelet reactivity than men (Ranucci et al., 2019). Together, concomitant development of atherosclerosis, endothelial dysfunction, and impairment of the coagulation profile lead to a favorable environment for IHD development in diabetic women (Figure 1).

Mechanisms involved in increased mortality following myocardial infarction in diabetic women are not fully understood. Nevertheless, energy metabolism has recently emerged to explain this sex difference (Figure 1). A strong decrease in ATP and phosphocreatine cardiac content has been observed following ischemia-reperfusion injury in prediabetic female rats fed with a high-fat, high-sucrose diet and in diabetic GK female rats (Fourny et al., 2019a, b). Importantly a previous study reports no difference in high-energy compounds following ischemia-reperfusion injury in male GK (Desrois et al., 2010), suggesting the important role of mitochondria and particularly the energy production pathway in female sensitivity to IHDs.

## Sexual Dimorphism in Heart Failure Associated With Diabetes

Heart failure is driven by CADs but also by aging, hypertension, diabetes, and obesity (Ho et al., 2016; Beale et al., 2018; Seferović et al., 2018). The excess risk of HF associated with diabetes is significantly greater in women with diabetes than in diabetic men (Ohkuma et al., 2019), increasing HF risk fivefold in women compared to 2.4-fold in men (Beale et al., 2018). Kim et al. (2019) also show that the impact of diabetes on long-term mortality and HF readmission seems to be greater in females than in males. Women represent ∼60% of patients having HF with preserved ejection fraction (HFpEF) whether they present with diabetes or not, but T2D women are younger, more obese, have worse renal function, lower prevalence of atrial fibrillation, and decreased hemoglobin levels (Lejeune et al., 2021). Importantly, HFpEF is more prevalent in women than in men, who preferentially exert HF with reduced ejection fraction (Beale et al., 2019; Dewan et al., 2019). In line, Weinberg et al. (1999) show sex-related differences in genes regulating calcium handling and contractile function. Males have higher beta-myosin heavy chain and atrial natriuretic factor, and lower SERCA-2 mRNAs in comparison with females despite a similar left ventricular hypertrophy and systolic overload (Weinberg et al., 1999).

Besides CADs and hypertension, the diabetic heart is characterized by alterations of its structure and mass as well as of its diastolic and systolic function, leading to the concept of DCM (Boudina and Abel, 2010). Interestingly, DCM prevalence is higher in women in comparison to men. In particular, Kiencke et al. (2010) show that female gender is an independent risk factor for DCM, characterized by greater structural and functional impairment (Kiencke et al., 2010; Toedebusch et al., 2018; Figure 1).

Myocardial remodeling occurs during DCM development with an increase in fibrosis and collagen I and III deposits, leading to myocardial rigidity (Murphy et al., 2017). Studies show greater myocardial remodeling and fibrosis in women with HF compared with men (Li et al., 2017). Women with T2D have greater cardiac hypertrophy, myocardial wall thickening, and an increase in left ventricular mass. Greater hypertrophy is also observed in female GK rats compared with males (Desrois et al., 2004). Estrogen receptor ERβ is shown to play an important role in the regulation of collagen levels (Schuster et al., 2016). Schuster et al. (2016) reveal that overexpression of ERβ in mice reduced myocardial fibrosis and collagen I/III deposits, improving cardiac function. Inversely, Skavdahl et al. (2005) detect basal cardiac hypertrophy in female mice deficient for ERβ, confirming the important role of this receptor for cardiac hypertrophy development in females. Moreover, imbalance between ERα and ERβ is reported in diabetic women and may explain the loss of estrogen cardioprotection regarding myocardial hypertrophy and fibrosis in DCM (Wells et al., 2005).

A fined-tuned regulation of metabolism and energy production is essential for heart function (Horman et al., 2012; Bertrand et al., 2020). Female cardiomyocytes contain less mitochondria, but they are more efficient than in male cardiomyocytes. Female hearts use fatty acids for energy production in greater proportion than males (Djouadi et al., 1998; Ventura-Clapier et al., 2017b). They also produce fewer ROS, have a lower calcium uptake rate, and a greater calcium retention capacity (Ventura-Clapier et al., 2017b). This sexual dimorphism does not lead to a difference in respiration and mitochondrial efficiency in the basal state but could play a role in pathological situations such as DCM. Of interest, a mitochondrial localization of estrogen receptors is reported, inducing direct effects of estrogen on mitochondrial respiration and antioxidant defenses (Gupte et al., 2015). Billimoria et al. (2006) show a greater mitochondrial respiration in female streptozotocin-treated (STZ) rats in comparison with corresponding males. Lagranha et al. (2010) show that the phosphorylation level of mitochondrial proteins is more important in females compared with males. This is particularly the case for the aldehyde dehydrogenase 2, leading to a decrease in ROS production (Lagranha et al., 2010; Tchernof and Despres, 2013).

Metabolic inflexibility is commonly noticed in the diabetic heart, which mainly relies on fatty acid oxidation for energy production (Vallerie and Bornfeldt, 2015). The increase in fatty acid oxidation in the diabetic heart is associated with an increase in PPARα, which plays a key role in the development of cardiac hypertrophy and dysfunction in DCM (Madrazo and Kelly, 2008; Bayeva et al., 2013). Interestingly, estrogen is involved in the signaling pathway of lipid metabolism and may explain the differences in mitochondrial metabolism observed between diabetic males and females. Indeed, Djouadi et al. (1998) show, in PPARα^–/–^ mice, that the subsequent inhibition of cellular fatty acid metabolism caused massive accumulation of hepatic and cardiac lipids, hypoglycemia, and death in 100% of males but only 25% of females. The treatment with β-estradiol decreased the mortality in males, demonstrating the role of female sex hormones in lipid homeostasis mediated by PPARα (Djouadi et al., 1998). In the last decade, micro-RNAs emerged as biomarkers of DCM and targets for new treatment. Yin et al. (2019) show that miR-30c protects cardiac metabolism and function in diabetes through PPARα modulation and its downstream effector, the co-activator Peroxisome proliferator-activated receptor gamma coactivator 1-alpha (PGC1α). The miR-208a, whose overexpression induced spontaneous cardiac hypertrophy (Callis et al., 2009), is another miR playing a role in DCM. Recently, Lum-Naihe et al. (2017) highlighted higher miR-208a expression in female diabetic hearts than in male counterparts.

## Personalized Care of Diabetic Patients

Involvement of female hormones in various physiological and pathophysiological processes has led the scientific community to focus their research on the male sex. However, we currently know that women have a different clinical presentation and drug response in multiple pathologies, including CVDs and T2D (Mathieu et al., 2018; Fourny et al., 2019b). First, clinical trials demonstrated sex differences in lifestyle intervention in diabetic patients. Moderate-intensity resistance exercise training is a more favorable approach for hypertensive women because of greater decreases in diastolic blood pressure and significant increases in flow-mediated dilation compared with their male counterparts (Collier et al., 2011). Weight loss with intensive lifestyle modification led to greater decreases in glucose/insulin concentration, insulin resistance, triglyceride, and glycated hemoglobin HbA1c levels in men than in women, indicating that women should particularly pay attention to risk factors, such as obesity (Perreault et al., 2008). This was confirmed in animal models in which diet change is most effective to reduce inflammation in male mice (Griffin et al., 2019).

Sex differences are also reported in regard to the response to antidiabetic drugs. In young patients, metformin plus rosiglitazone was more effective in girls than in boys (Zeitler et al., 2012). In adults, women had a higher reduction of body weight after treatment with metformin or sulfonylurea, whereas men displayed higher HbA1c reduction after treatment with metformin only (Schütt et al., 2015). Sex differences were also reported for incretins with a better glycemic control in men (Anichini et al., 2013) while others showed greater weight loss, reductions of fasting glucose, and blood pressure levels in women (Pencek et al., 2012). The LEADER study highlighted a greater CV benefit in men than in women with liraglutide treatment (Verma et al., 2018). Recently, Raparelli et al. (2020) demonstrated greater CV effectiveness of GLP-1 receptor agonist in women. However, “the real-world experience” study showed that men achieved target glycemic response in higher proportions than women after 1 year of exenatide (Anichini et al., 2013). A greater glycemic response and HbA1c reduction was found with sulfonylureas than with thiazolidinediones in men, whereas female sex was associated with greater HbA1c reduction but a weight gain and edema risk with thiazolidinediones (Dennis et al., 2018). Interestingly, Zinman et al. (2015) reported no sex difference in the EMPA-REG OUTCOME trial in effects of a sodium-glucose cotransporter 2 inhibitor. However, subgroup analysis showed a significant CV benefit in males only (Kautzky-Willer and Harreiter, 2017). In 2020, an important study showed no differences for vascular efficacy outcomes or death with major protection against major adverse CV events, HF, vascular death, and total mortality in both men and women (Rådholm et al., 2020). Taken together, these studies clearly show that sex should be considered in the choice of antidiabetic treatment to move toward “precision medicine,” which aims to treat patients with accurate care that is more personalized and including individual variability (Currie and Delles, 2018; Prasad and Groop, 2019). However, mechanisms involved in these differences are not yet understood, and disparity of antidiabetic treatments used, alone or in combination, makes comparison difficult.

The choice of the animal model to be employed is also delicate for the efficient transfer of results to humans, particularly when comparing males and females. Indeed, studying females is not always possible in animal T2D models. For example, the female TallyHo mouse does not show hyperglycemia unlike males (Kim et al., 2005) and the female Nagoya-Shibata-Yasuda mouse has a low incidence of type 2 diabetes compared with males (Ueda et al., 1995). Besides this, enriched diets are also commonly used in the literature, but their diversity and duration make comparison difficult. Thus, each diet-induced and energetic diabetic model should be well characterized to ensure good interpretation of the results obtained in both sexes.

## Conclusion

In conclusion, clinical and animal studies clearly indicate that there are sex differences in T2D-associated CV complications. However, the precise molecular mechanisms responsible for these differences are still largely blurred. Recent studies have particularly emphasized the link between energy metabolism and miRs. Thus, future studies should particularly pay attention to the metabolic dysfunctions that are involved in both IHDs and DCM development. This could provide new targets for the treatment of the diabetic heart. In addition, the antidiabetic drug response also differs significantly according to sex. Therefore, the scientific community must include both sexes in future clinical trials and animal studies to improve quality of care and bring a more personalized treatment to each patient.

## Author Contributions

All authors listed have made a substantial, direct and intellectual contribution to the work, and approved it for publication.

## Conflict of Interest

The authors declare that the research was conducted in the absence of any commercial or financial relationships that could be construed as a potential conflict of interest.

## References

[B1] Alameddine A. Fajloun Z. Bourreau J. Gauquelin-Koch G. Yuan M. Gauguier D. (2015). The cardiovascular effects of salidroside in the Goto-Kakizaki diabetic rat model. *J. Physiol. Pharmacol.* 66 249–257. 25903955

[B2] American Diabetes Association (2015). (8) Cardiovascular disease and risk management. *Diabetes Care* 38 Suppl S49–S57. 25537708 10.2337/dc15-S011

[B3] Anand S. S. Islam S. Rosengren A. Franzosi M. G. Steyn K. Yusufali A. H. (2008). Risk factors for myocardial infarction in women and men: insights from the INTERHEART study. *Eur. Heart J.* 29 932–940. 10.1093/eurheartj/ehn018 18334475

[B4] Anichini R. Cosimi S. Di Carlo A. Orsini P. De Bellis A. Seghieri G. (2013). Gender difference in response predictors after 1-year exenatide therapy twice daily in type 2 diabetic patients: a real world experience. *Diabetes Metab. Syndr. Obes.* 6 123–129. 10.2147/dmso.s42729 23630427 PMC3626369

[B5] Bayeva M. Sawicki K. T. Ardehali H. (2013). Taking diabetes to heart–deregulation of myocardial lipid metabolism in diabetic cardiomyopathy. *J. Am. Heart Assoc.* 2:e000433. 24275630 10.1161/JAHA.113.000433 PMC3886738

[B6] Beale A. L. Meyer P. Marwick T. H. Lam C. S. P. Kaye D. M. (2018). Sex differences in cardiovascular pathophysiology: why women are overrepresented in heart failure with preserved ejection fraction. *Circulation* 138 198–205. 10.1161/circulationaha.118.034271 29986961

[B7] Beale A. L. Nanayakkara S. Segan L. Mariani J. A. Maeder M. T. van Empel V. (2019). Sex differences in heart failure with preserved ejection fraction pathophysiology: a detailed invasive hemodynamic and echocardiographic analysis. *JACC Heart Fail.* 7 239–249. 30819380 10.1016/j.jchf.2019.01.004

[B8] Bertrand L. Auquier J. Renguet E. Angé M. Cumps J. Horman S. (2020). Glucose transporters in cardiovascular system in health and disease. *Pflugers. Arch.* 472 1385–1399. 32809061 10.1007/s00424-020-02444-8

[B9] Billimoria F. R. Katyare S. S. Patel S. P. (2006). Insulin status differentially affects energy transduction in cardiac mitochondria from male and female rats. *Diabetes Obes Metab.* 8 67–74. 10.1111/j.1463-1326.2005.00470.x 16367884

[B10] Blenck C. L. Harvey P. A. Reckelhoff J. F. Leinwand L. A. (2016). The importance of biological sex and estrogen in rodent models of cardiovascular health and disease. *Circ. Res.* 118 1294–1312. 10.1161/circresaha.116.307509 27081111 PMC4834858

[B11] Blüher M. (2013). Importance of estrogen receptors in adipose tissue function. *Mol. Metab.* 2 130–132. 10.1016/j.molmet.2013.07.001 24049727 PMC3773840

[B12] Boudina S. Abel E. D. (2010). Diabetic cardiomyopathy, causes and effects. *Rev. Endocr. Metab. Disord.* 11 31–39. 10.1007/s11154-010-9131-7 20180026 PMC2914514

[B13] Brinkley T. E. Kume N. Mitsuoka H. Phares D. A. Hagberg J. M. (2008). Elevated soluble lectin-like oxidized LDL receptor-1 (sLOX-1) levels in obese postmenopausal women. *Obesity (Silver Spring)* 16 1454–1456. 10.1038/oby.2008.213 18388896 PMC2677801

[B14] Bugger H. Abel E. D. (2014). Molecular mechanisms of diabetic cardiomyopathy. *Diabetologia* 57 660–671. 10.1007/s00125-014-3171-6 24477973 PMC3969857

[B15] Bugiardini R. Ricci B. Cenko E. Vasiljevic Z. Kedev S. Davidovic G. (2017). Delayed care and mortality among women and men with myocardial infarction. *J. Am. Heart Assoc.* 6:e005968. 28862963 10.1161/JAHA.117.005968 PMC5586439

[B16] Callis T. E. Pandya K. Seok H. Y. Tang R. H. Tatsuguchi M. Huang Z. P. (2009). MicroRNA-208a is a regulator of cardiac hypertrophy and conduction in mice. *J. Clin. Invest.* 119 2772–2786. 19726871 10.1172/JCI36154 PMC2735902

[B17] Chen M. Masaki T. Sawamura T. (2002). LOX-1, the receptor for oxidized low-density lipoprotein identified from endothelial cells: implications in endothelial dysfunction and atherosclerosis. *Pharmacol. Ther.* 95 89–100. 10.1016/s0163-7258(02)00236-x 12163130

[B18] Cid M. C. Schnaper H. W. Kleinman H. K. (2002). Estrogens and the vascular endothelium. *Ann. N. Y. Acad. Sci.* 966 143–157. 10.1111/j.1749-6632.2002.tb04211.x 12114268

[B19] Collier S. R. Frechette V. Sandberg K. Schafer P. Ji H. Smulyan H. (2011). Sex differences in resting hemodynamics and arterial stiffness following 4 weeks of resistance versus aerobic exercise training in individuals with pre-hypertension to stage 1 hypertension. *Biol. Sex Differ.* 2:9. 10.1186/2042-6410-2-9 21867499 PMC3184039

[B20] Currie G. Delles C. (2018). Precision medicine and personalized medicine in cardiovascular disease. *Adv. Exp. Med. Biol.* 1065 589–605. 10.1007/978-3-319-77932-4_36 30051409

[B21] Dantas A. P. Fortes Z. B. de Carvalho M. H. (2012). Vascular disease in diabetic women: why do they miss the female protection? *Exp. Diabetes Res.* 2012:570598. 22973304 10.1155/2012/570598 PMC3438753

[B22] Davis K. E. Neinast M. D. Sun K. Skiles W. M. Bills J. D. Zehr J. A. (2013). The sexually dimorphic role of adipose and adipocyte estrogen receptors in modulating adipose tissue expansion, inflammation, and fibrosis. *Mol. Metab.* 2 227–242. 10.1016/j.molmet.2013.05.006 24049737 PMC3773827

[B23] Dennis J. M. Henley W. E. Weedon M. N. Lonergan M. Rodgers L. R. Jones A. G. (2018). Sex and BMI alter the benefits and risks of sulfonylureas and thiazolidinediones in type 2 diabetes: a framework for evaluating stratification using routine clinical and individual trial data. *Diabetes Care* 41 1844–1853. 10.2337/dc18-0344 30072404 PMC6591127

[B24] Desrois M. Clarke K. Lan C. Dalmasso C. Cole M. Portha B. (2010). Upregulation of eNOS and unchanged energy metabolism in increased susceptibility of the aging type 2 diabetic GK rat heart to ischemic injury. *Am. J. Physiol. Heart Circ. Physiol.* 299 H1679–H1686. 20729402 10.1152/ajpheart.00998.2009 PMC2993220

[B25] Desrois M. Lan C. Movassat J. Bernard M. (2017). Reduced up-regulation of the nitric oxide pathway and impaired endothelial and smooth muscle functions in the female type 2 diabetic goto-kakizaki rat heart. *Nutr. Metab. (Lond.)* 14:6. 28101124 10.1186/s12986-016-0157-z PMC5237314

[B26] Desrois M. Sidell R. J. Gauguier D. Davey C. L. Radda G. K. Clarke K. (2004). Gender differences in hypertrophy, insulin resistance and ischemic injury in the aging type 2 diabetic rat heart. *J. Mol. Cell Cardiol.* 37 547–555. 10.1016/j.yjmcc.2004.05.014 15276024

[B27] Dewan P. Rørth R. Raparelli V. Campbell R. T. Shen L. Jhund P. S. (2019). Sex-related differences in heart failure with preserved ejection fraction. *Circ. Heart Fail.* 12:e006539. 31813280 10.1161/CIRCHEARTFAILURE.119.006539

[B28] Djouadi F. Weinheimer C. J. Saffitz J. E. Pitchford C. Bastin J. Gonzalez F. J. (1998). A gender-related defect in lipid metabolism and glucose homeostasis in peroxisome proliferator- activated receptor alpha- deficient mice. *J. Clin. Invest.* 102 1083–1091. 10.1172/jci3949 9739042 PMC509091

[B29] Donahue R. P. Dorn J. M. Stranges S. Swanson M. Hovey K. Trevisan M. (2011). Impaired fasting glucose and recurrent cardiovascular disease among survivors of a first acute myocardial infarction: evidence of a sex difference? The Western New York experience. *Nutr. Metab. Cardiovasc. Dis.* 21 504–511. 10.1016/j.numecd.2009.11.012 20227262 PMC2888844

[B30] Donahue R. P. Rejman K. Rafalson L. B. Dmochowski J. Stranges S. Trevisan M. (2007). Sex differences in endothelial function markers before conversion to pre-diabetes: does the clock start ticking earlier among women? The Western New York Study. *Diabetes Care* 30 354–359. 10.2337/dc06-1772 17259507

[B31] Elffers T. W. de Mutsert R. Lamb H. J. de Roos A. Willems van Dijk K. Rosendaal F. R. (2017). Body fat distribution, in particular visceral fat, is associated with cardiometabolic risk factors in obese women. *PLoS One* 12:e0185403. 10.1371/journal.pone.0185403 28957363 PMC5619737

[B32] Eshak E. S. Iso H. Mizoue T. Inoue M. Noda M. Tsugane S. (2013). Soft drink, 100% fruit juice, and vegetable juice intakes and risk of diabetes mellitus. *Clin. Nutr.* 32 300–308. 10.1016/j.clnu.2012.08.003 22917499

[B33] Flanagan D. E. Holt R. I. Owens P. C. Cockington R. J. Moore V. M. Robinson J. S. (2006). Gender differences in the insulin-like growth factor axis response to a glucose load. *Acta Physiol. (Oxf.)* 187 371–378. 10.1111/j.1748-1716.2006.01581.x 16776662

[B34] Fourny N. Lan C. Kober F. Boulghobra D. Bresciani J. Reboul C. (2019a). Cardiac remodeling and higher sensitivity to ischemia-reperfusion injury in female rats submitted to high-fat high-sucrose diet: an in vivo/ex vivo longitudinal follow-up. *J. Nutr. Biochem.* 69 139–150. 10.1016/j.jnutbio.2019.03.022 31082660

[B35] Fourny N. Lan C. Sérée E. Bernard M. Desrois M. (2019b). Protective effect of resveratrol against ischemia-reperfusion injury via enhanced high energy compounds and eNOS-SIRT1 expression in type 2 diabetic female rat heart. *Nutrients* 11:105. 10.3390/nu11010105 30621358 PMC6356423

[B36] Garcia M. Mulvagh S. L. Merz C. N. Buring J. E. Manson J. E. (2016). Cardiovascular disease in women: clinical perspectives. *Circ. Res.* 118 1273–1293. 10.1161/circresaha.116.307547 27081110 PMC4834856

[B37] Goel A. Thor D. Anderson L. Rahimian R. (2008). Sexual dimorphism in rabbit aortic endothelial function under acute hyperglycemic conditions and gender-specific responses to acute 17beta-estradiol. *Am. J. Physiol. Heart Circ. Physiol.* 294 H2411–H2420. 18326804 10.1152/ajpheart.01217.2007

[B38] Griffin C. Hutch C. R. Abrishami S. Stelmak D. Eter L. Li Z. (2019). Inflammatory responses to dietary and surgical weight loss in male and female mice. *Biol. Sex Differ.* 10:16. 30944030 10.1186/s13293-019-0229-7 PMC6446331

[B39] Gupte A. A. Pownall H. J. Hamilton D. J. (2015). Estrogen: an emerging regulator of insulin action and mitochondrial function. *J. Diabetes Res.* 2015:916585. 25883987 10.1155/2015/916585 PMC4391691

[B40] Harrington J. Fillmore N. Gao S. Yang Y. Zhang X. Liu P. (2017). A systems biology approach to investigating sex differences in cardiac hypertrophy. *J. Am. Heart Assoc.* 6:e005838. 28862954 10.1161/JAHA.117.005838 PMC5586433

[B41] Ho J. E. Enserro D. Brouwers F. P. Kizer J. R. Shah S. J. Psaty B. M. (2016). Predicting heart failure with preserved and reduced ejection fraction: the international collaboration on heart failure subtypes. *Circ. Heart Fail.* 9:e003116. 27266854 10.1161/CIRCHEARTFAILURE.115.003116 PMC4902276

[B42] Horman S. Beauloye C. Vanoverschelde J. L. Bertrand L. (2012). AMP-activated protein kinase in the control of cardiac metabolism and remodeling. *Curr. Heart Fail. Rep.* 9 164–173. 10.1007/s11897-012-0102-z 22767403

[B43] Huang D. Refaat M. Mohammedi K. Jayyousi A. Al Suwaidi J. Abi Khalil C. (2017). Macrovascular complications in patients with diabetes and prediabetes. *Biomed. Res. Int.* 2017:7839101. 29238721 10.1155/2017/7839101 PMC5697393

[B44] Hunter I. Soler A. Joseph G. Hutcheson B. Bradford C. Zhang F. F. (2017). Cardiovascular function in male and female JCR:LA-cp rats: effect of high-fat/high-sucrose diet. *Am. J. Physiol. Heart Circ. Physiol.* 312 H742–H751. 28087518 10.1152/ajpheart.00535.2016 PMC5407169

[B45] International Diabetes Federation (2019). *IDF Diabetes Atlas, t.e.B.* Brussels: International Diabetes Federation.

[B46] Iorga A. Cunningham C. M. Moazeni S. Ruffenach G. Umar S. Eghbali M. (2017). The protective role of estrogen and estrogen receptors in cardiovascular disease and the controversial use of estrogen therapy. *Biol. Sex Differ.* 8:33. 29065927 10.1186/s13293-017-0152-8 PMC5655818

[B47] Iozzo P. Lautamaki R. Borra R. Lehto H. R. Bucci M. Viljanen A. (2009). Contribution of glucose tolerance and gender to cardiac adiposity. *J. Clin. Endocrinol. Metab.* 94 4472–4482. 10.1210/jc.2009-0436 19820028

[B48] Kander M. C. Cui Y. Liu Z. (2017). Gender difference in oxidative stress: a new look at the mechanisms for cardiovascular diseases. *J. Cell Mol. Med.* 21 1024–1032. 10.1111/jcmm.13038 27957792 PMC5387169

[B49] Kannel W. B. (1987). Metabolic risk factors for coronary heart disease in women: perspective from the Framingham study. *Am. Heart J.* 114 413–419. 10.1016/0002-8703(87)90511-4 3604900

[B50] Kannel W. B. Hjortland M. Castelli W. P. (1974). Role of diabetes in congestive heart failure: the Framingham study. *Am. J. Cardiol.* 34 29–34. 10.1016/0002-9149(74)90089-7 4835750

[B51] Kannel W. B. McGee D. L. (1979). Diabetes and cardiovascular disease. The Framingham study. *JAMA* 241 2035–2038. 10.1001/jama.1979.03290450033020 430798

[B52] Kararigas G. Dworatzek E. Petrov G. Summer H. Schulze T. M. Baczko I. (2014). Sex-dependent regulation of fibrosis and inflammation in human left ventricular remodelling under pressure overload. *Eur. J. Heart Fail.* 16 1160–1167. 10.1002/ejhf.171 25287281

[B53] Kautzky-Willer A. Harreiter J. (2017). Sex and gender differences in therapy of type 2 diabetes. *Diabetes Res. Clin. Pract.* 131 230–241. 10.1016/j.diabres.2017.07.012 28779681

[B54] Kautzky-Willer A. Harreiter J. Pacini G. (2016). Sex and gender differences in risk, pathophysiology and complications of type 2 diabetes mellitus. *Endocr. Rev.* 37 278–316. 10.1210/er.2015-1137 27159875 PMC4890267

[B55] Kiencke S. Handschin R. von Dahlen R. Muser J. Brunner-Larocca H. P. Schumann J. (2010). Pre-clinical diabetic cardiomyopathy: prevalence, screening, and outcome. *Eur. J. Heart Fail.* 12 951–957. 10.1093/eurjhf/hfq110 20581103

[B56] Kim H. L. Kim M. A. Park K. T. Choi D. J. Han S. Jeon E. S. (2019). Gender difference in the impact of coexisting diabetes mellitus on long-term clinical outcome in people with heart failure: a report from the Korean Heart Failure Registry. *Diabet. Med.* 36 1312–1318. 10.1111/dme.14059 31254366

[B57] Kim J. H. Stewart T. P. Zhang W. Kim H. Y. Nishina P. M. Naggert J. K. (2005). Type 2 diabetes mouse model TallyHo carries an obesity gene on chromosome 6 that exaggerates dietary obesity. *Physiol. Genomics* 22 171–181. 10.1152/physiolgenomics.00197.2004 15870394

[B58] Kjeldsen S. E. (2018). Hypertension and cardiovascular risk: general aspects. *Pharmacol Res.* 129 95–99. 10.1016/j.phrs.2017.11.003 29127059

[B59] Koro C. E. Bowlin S. J. Rabatin V. Fedder D. O. (2008). Cardiovascular disease risk among subjects with impaired fasting glucose in the United States: results from NHANES 1999–2004. *Diabetes Metab. Syndr.* 2 239–244. 10.1016/j.dsx.2008.07.003

[B60] Kriska A. M. Edelstein S. L. Hamman R. F. Otto A. Bray G. A. Mayer-Davis E. J. (2006). Physical activity in individuals at risk for diabetes: diabetes prevention program. *Med. Sci. Sports Exerc.* 38 826–832. 10.1249/01.mss.0000218138.91812.f9 16672833 PMC1570396

[B61] Krumholz H. M. Larson M. Levy D. (1993). Sex differences in cardiac adaptation to isolated systolic hypertension. *Am. J. Cardiol.* 72 310–313. 10.1016/0002-9149(93)90678-6 8342510

[B62] Lagranha C. J. Deschamps A. Aponte A. Steenbergen C. Murphy E. (2010). Sex differences in the phosphorylation of mitochondrial proteins result in reduced production of reactive oxygen species and cardioprotection in females. *Circ. Res.* 106 1681–1691. 10.1161/circresaha.109.213645 20413785 PMC3127199

[B63] Lejeune S. Roy C. Slimani A. Pasquet A. Vancraeynest D. Vanoverschelde J. L. (2021). Diabetic phenotype and prognosis of patients with heart failure and preserved ejection fraction in a real life cohort. *Cardiovasc. Diabetol.* 20:48. 33608002 10.1186/s12933-021-01242-5 PMC7893869

[B64] Levitzky Y. S. Pencina M. J. D’Agostino R. B. Meigs J. B. Murabito J. M. Vasan R. S. (2008). Impact of impaired fasting glucose on cardiovascular disease: the Framingham heart study. *J. Am. Coll. Cardiol.* 51 264–270. 18206734 10.1016/j.jacc.2007.09.038

[B65] Li Z. Wang Z. Yin Z. Zhang Y. Xue X. Han J. (2017). Gender differences in fibrosis remodeling in patients with long-standing persistent atrial fibrillation. *Oncotarget* 8 53714–53729. 10.18632/oncotarget.16342 28881845 PMC5581144

[B66] Lind L. Ärnlöv J. Lampa E. (2017). The interplay between fat mass and fat distribution as determinants of the metabolic syndrome is sex-dependent. *Metab. Syndr. Relat. Disord.* 15 337–343. 10.1089/met.2017.0006 28586263

[B67] Listenberger L. L. Ory D. S. Schaffer J. E. (2001). Palmitate-induced apoptosis can occur through a ceramide-independent pathway. *J. Biol. Chem.* 276 14890–14895. 10.1074/jbc.m010286200 11278654

[B68] Lum-Naihe K. Toedebusch R. Mahmood A. Bajwa J. Carmack T. Kumar S. A. (2017). Cardiovascular disease progression in female Zucker Diabetic Fatty rats occurs via unique mechanisms compared to males. *Sci. Rep.* 7:17823. 29259233 10.1038/s41598-017-18003-8 PMC5736602

[B69] Madhu S. V. (2010). Endothelial dysfunction and diabetes. *J. Assoc. Physicians India* 58 475–476. 21189692

[B70] Madrazo J. A. Kelly D. P. (2008). The PPAR trio: regulators of myocardial energy metabolism in health and disease. *J. Mol. Cell Cardiol.* 44 968–975. 10.1016/j.yjmcc.2008.03.021 18462747

[B71] Mathieu C. Desrois M. Kober F. Lalevée N. Lan C. Fourny N. (2018). Sex-mediated response to the beta-blocker landiolol in sepsis: an experimental, randomized study. *Crit. Care Med.* 46 e684–e691. 29634521 10.1097/CCM.0000000000003146

[B72] Mehta L. S. Beckie T. M. DeVon H. A. Grines C. L. Krumholz H. M. Johnson M. N. (2016). Acute myocardial infarction in women: a scientific statement from the american heart association. *Circulation* 133 916–947. 10.1161/cir.0000000000000351 26811316

[B73] Meigs J. B. O’Donnell C. J. Tofler G. H. Benjamin E. J. Fox C. S. Lipinska I. (2006). Hemostatic markers of endothelial dysfunction and risk of incident type 2 diabetes: the Framingham offspring study. *Diabetes* 55 530–537. 10.2337/diabetes.55.02.06.db05-1041 16443791

[B74] Murphy E. (2011). Estrogen signaling and cardiovascular disease. *Circ. Res.* 109 687–696. 10.1161/circresaha.110.236687 21885836 PMC3398381

[B75] Murphy E. Amanakis G. Fillmore N. Parks R. J. Sun J. (2017). Sex differences in metabolic cardiomyopathy. *Cardiovasc. Res.* 113 370–377. 10.1093/cvr/cvx008 28158412 PMC5852638

[B76] Natarajan S. Liao Y. Sinha D. Cao G. McGee D. L. Lipsitz S. R. (2005). Sex differences in the effect of diabetes duration on coronary heart disease mortality. *Arch. Intern. Med.* 165 430–435. 10.1001/archinte.165.4.430 15738373

[B77] Ohkuma T. Komorita Y. Peters S. A. E. Woodward M. (2019). Diabetes as a risk factor for heart failure in women and men: a systematic review and meta-analysis of 47 cohorts including 12 million individuals. *Diabetologia* 62 1550–1560. 10.1007/s00125-019-4926-x 31317230 PMC6677875

[B78] Olsson M. C. Palmer B. M. Leinwand L. A. Moore R. L. (2001). Gender and aging in a transgenic mouse model of hypertrophic cardiomyopathy. *Am. J. Physiol. Heart Circ. Physiol.* 280 H1136–H1144. 11179057 10.1152/ajpheart.2001.280.3.H1136

[B79] Palee S. Minta W. Mantor D. Sutham W. Pratchayasakul W. Chattipakorn S. (2017). Estrogen deprivation aggravates cardiometabolic dysfunction and intracellular calcium dyshomeostasis in obese-insulin resistance rats. *J. Am. Coll. Cardiol.* 69:681. 10.1016/s0735-1097(17)34070-6

[B80] Pencek R. Blickensderfer A. Li Y. Brunell S. C. Chen S. (2012). Exenatide once weekly for the treatment of type 2 diabetes: effectiveness and tolerability in patient subpopulations. *Int. J. Clin. Pract.* 66 1021–1032. 10.1111/j.1742-1241.2012.03006.x 22925173 PMC3506736

[B81] Perreault L. Ma Y. Dagogo-Jack S. Horton E. Marrero D. Crandall J. (2008). Sex differences in diabetes risk and the effect of intensive lifestyle modification in the diabetes prevention program. *Diabetes Care* 31 1416–1421. 10.2337/dc07-2390 18356403 PMC2453677

[B82] Power M. L. Schulkin J. (2008). Sex differences in fat storage, fat metabolism, and the health risks from obesity: possible evolutionary origins. *Br. J. Nutr.* 99 931–940. 10.1017/s0007114507853347 17977473

[B83] Prasad R. B. Groop L. (2019). Precision medicine in type 2 diabetes. *J. Intern. Med.* 285 40–48. 10.1111/joim.12859 30403316

[B84] Rådholm K. Zhou Z. Clemens K. Neal B. Woodward M. (2020). Effects of sodium-glucose co-transporter-2 inhibitors in type 2 diabetes in women versus men. *Diabetes Obes. Metab.* 22 263–266. 10.1111/dom.13876 31486272

[B85] Ranucci M. Aloisio T. Di Dedda U. Menicanti L. de Vincentiis C. Baryshnikova E. (2019). Gender-based differences in platelet function and platelet reactivity to P2Y12 inhibitors. *PLoS One* 14:e0225771. 10.1371/journal.pone.0225771 31774869 PMC6881030

[B86] Raparelli V. Elharram M. Moura C. S. Abrahamowicz M. Bernatsky S. Behlouli H. (2020). Sex differences in cardiovascular effectiveness of newer glucose-lowering drugs added to metformin in type 2 diabetes mellitus. *J. Am. Heart Assoc.* 9:e012940. 31902326 10.1161/JAHA.119.012940 PMC6988160

[B87] Reichelt M. E. Mellor K. M. Bell J. R. Chandramouli C. Headrick J. P. Delbridge L. M. (2013). Sex, sex steroids, and diabetic cardiomyopathy: making the case for experimental focus. *Am. J. Physiol. Heart Circ. Physiol.* 305 H779–H792. 23792676 10.1152/ajpheart.00141.2013

[B88] Rutter M. K. Parise H. Benjamin E. J. Levy D. Larson M. G. Meigs J. B. (2003). Impact of glucose intolerance and insulin resistance on cardiac structure and function: sex-related differences in the Framingham heart study. *Circulation* 107 448–454. 10.1161/01.cir.0000045671.62860.98 12551870

[B89] Rydén L. Standl E. Bartnik M. Van den Berghe G. Betteridge J. de Boer M. J. (2007). Guidelines on diabetes, pre-diabetes, and cardiovascular diseases: executive summary. The Task Force on Diabetes and Cardiovascular Diseases of the European Society of Cardiology (ESC) and of the European Association for the Study of Diabetes (EASD). *Eur. Heart J.* 28 88–136. 17220161 10.1093/eurheartj/ehl260

[B90] Saengsirisuwan V. Pongseeda S. Prasannarong M. Vichaiwong K. Toskulkao C. (2009). Modulation of insulin resistance in ovariectomized rats by endurance exercise training and estrogen replacement. *Metabolism* 58 38–47. 10.1016/j.metabol.2008.08.004 19059529

[B91] Santos M. Shah A. M. (2014). Alterations in cardiac structure and function in hypertension. *Curr. Hypertens Rep.* 16:428. 24639061 10.1007/s11906-014-0428-x PMC4051201

[B92] Schuster I. Mahmoodzadeh S. Dworatzek E. Jaisser F. Messaoudi S. Morano I. (2016). Cardiomyocyte-specific overexpression of oestrogen receptor β improves survival and cardiac function after myocardial infarction in female and male mice. *Clin. Sci. (Lond.)* 130 365–376. 10.1042/cs20150609 26608078

[B93] Schütt M. Zimmermann A. Hood R. Hummel M. Seufert J. Siegel E. (2015). Gender-specific effects of treatment with lifestyle, metformin or sulfonylurea on glycemic control and body weight: a german multicenter analysis on 9 108 patients. *Exp. Clin. Endocrinol. Diabetes* 123 622–626. 10.1055/s-0035-1559608 26285070

[B94] Seferović P. M. Petrie M. C. Filippatos G. S. Anker S. D. Rosano G. Bauersachs J. (2018). Type 2 diabetes mellitus and heart failure: a position statement from the Heart Failure Association of the European Society of Cardiology. *Eur. J. Heart Fail.* 20 853–872. 29520964 10.1002/ejhf.1170

[B95] Skavdahl M. Steenbergen C. Clark J. Myers P. Demianenko T. Mao L. (2005). Estrogen receptor-beta mediates male-female differences in the development of pressure overload hypertrophy. *Am. J. Physiol. Heart Circ. Physiol.* 288 H469–H476. 15374829 10.1152/ajpheart.00723.2004

[B96] Steinberg H. O. Paradisi G. Cronin J. Crowde K. Hempfling A. Hook G. (2000). Type II diabetes abrogates sex differences in endothelial function in premenopausal women. *Circulation* 101 2040–2046. 10.1161/01.cir.101.17.2040 10790344

[B97] Tchernof A. Despres J. P. (2013). Pathophysiology of human visceral obesity: an update. *Physiol. Rev.* 93 359–404. 10.1152/physrev.00033.2011 23303913

[B98] Thom T. Haase N. Rosamond W. Howard V. J. Rumsfeld J. Manolio T. (2006). Heart disease and stroke statistics–2006 update: a report from the American Heart Association Statistics Committee and Stroke Statistics Subcommittee. *Circulation* 113 e85–e151. 16407573 10.1161/CIRCULATIONAHA.105.171600

[B99] Thomas D. (2017). [Cardiovascular risk of smoking by gender]. *Presse. Med.* 46 681–687. 28619581 10.1016/j.lpm.2017.05.026

[B100] Toedebusch R. Belenchia A. Pulakat L. (2018). Diabetic cardiomyopathy: impact of biological sex on disease development and molecular signatures. *Front. Physiol.* 9:453. 10.3389/fphys.2018.00453 29773993 PMC5943496

[B101] Ueda H. Ikegami H. Yamato E. Fu J. Fukuda M. Shen G. (1995). The NSY mouse: a new animal model of spontaneous NIDDM with moderate obesity. *Diabetologia* 38 503–508. 10.1007/s001250050312 7489831

[B102] Vallerie S. N. Bornfeldt K. E. (2015). Metabolic flexibility and dysfunction in cardiovascular cells. *Arterioscler. Thromb. Vasc. Biol.* 35 e37–e42. 26310811 10.1161/ATVBAHA.115.306226 PMC4555874

[B103] Ventura-Clapier R. Dworatzek E. Seeland U. Kararigas G. Arnal J. F. Brunelleschi S. (2017a). Sex in basic research: concepts in the cardiovascular field. *Cardiovasc. Res.* 113 711–724. 10.1093/cvr/cvx066 28472454

[B104] Ventura-Clapier R. Moulin M. Piquereau J. Lemaire C. Mericskay M. Veksler V. (2017b). Mitochondria: a central target for sex differences in pathologies. *Clin. Sci. (Lond.)* 131 803–822. 10.1042/cs20160485 28424375

[B105] Verma S. Poulter N. R. Bhatt D. L. Bain S. C. Buse J. B. Leiter L. A. (2018). Effects of liraglutide on cardiovascular outcomes in patients with type 2 diabetes mellitus with or without history of myocardial infarction or stroke. *Circulation* 138 2884–2894. 30566004 10.1161/CIRCULATIONAHA.118.034516

[B106] Walden C. E. Knopp R. H. Wahl P. W. Beach K. W. Strandness E. Jr. (1984). Sex differences in the effect of diabetes mellitus on lipoprotein triglyceride and cholesterol concentrations. *N. Engl. J. Med.* 311 953–959. 10.1056/nejm198410113111505 6472421

[B107] Wannamethee S. G. Papacosta O. Lawlor D. A. Whincup P. H. Lowe G. D. Ebrahim S. (2012). Do women exhibit greater differences in established and novel risk factors between diabetes and non-diabetes than men? The British Regional Heart Study and British Women’s Heart Health Study. *Diabetologia* 55 80–87. 10.1007/s00125-011-2284-4 21861177

[B108] Weinberg E. O. Thienelt C. D. Katz S. E. Bartunek J. Tajima M. Rohrbach S. (1999). Gender differences in molecular remodeling in pressure overload hypertrophy. *J. Am. Coll. Cardiol.* 34 264–273. 10.1016/s0735-1097(99)00165-5 10400020

[B109] Wells C. C. Riazi S. Mankhey R. W. Bhatti F. Ecelbarger C. Maric C. (2005). Diabetic nephropathy is associated with decreased circulating estradiol levels and imbalance in the expression of renal estrogen receptors. *Gend. Med.* 2 227–237. 10.1016/s1550-8579(05)80052-x 16464734

[B110] Willi C. Bodenmann P. Ghali W. A. Faris P. D. Cornuz J. (2007). Active smoking and the risk of type 2 diabetes: a systematic review and meta-analysis. *JAMA* 298 2654–2664. 10.1001/jama.298.22.2654 18073361

[B111] Xing D. Nozell S. Chen Y. F. Hage F. Oparil S. (2009). Estrogen and mechanisms of vascular protection. *Arterioscler. Thromb. Vasc. Biol.* 29 289–295. 10.1161/atvbaha.108.182279 19221203 PMC2700771

[B112] Yang Y. Wang I. W. Turrentine M. Wang M. (2018). Postischemic application of estrogen ameliorates myocardial damage in an in vivo mouse model. *J. Surg. Res.* 231 366–372. 10.1016/j.jss.2018.05.076 30278955

[B113] Yin Z. Zhao Y. He M. Li H. Fan J. Nie X. (2019). MiR-30c/PGC-1β protects against diabetic cardiomyopathy via PPARα. *Cardiovasc. Diabetol.* 18:7. 30635067 10.1186/s12933-019-0811-7 PMC6329097

[B114] Zeitler P. Hirst K. Pyle L. Linder B. Copeland K. Arslanian S. (2012). A clinical trial to maintain glycemic control in youth with type 2 diabetes. *N. Engl. J. Med.* 366 2247–2256. 10.1056/nejmoa1109333 22540912 PMC3478667

[B115] Zhang R. Thor D. Han X. Anderson L. Rahimian R. (2012). Sex differences in mesenteric endothelial function of streptozotocin-induced diabetic rats: a shift in the relative importance of EDRFs. *Am. J. Physiol. Heart Circ. Physiol.* 303 H1183–H1198. 22982780 10.1152/ajpheart.00327.2012 PMC3517641

[B116] Zinman B. Wanner C. Lachin J. M. Fitchett D. Bluhmki E. Hantel S. (2015). Empagliflozin, cardiovascular outcomes, and mortality in type 2 diabetes. *N. Engl. J. Med.* 373 2117–2128. 26378978 10.1056/NEJMoa1504720

